# Apparent Acquired Resistance by a Weevil to Its Parasitoid Is Influenced by Host Plant

**DOI:** 10.3389/fpls.2016.01259

**Published:** 2016-08-23

**Authors:** Stephen L. Goldson, Federico Tomasetto

**Affiliations:** ^1^AgResearch Ltd.Christchurch, New Zealand; ^2^Bio-Protection Research Centre, Lincoln UniversityLincoln, New Zealand

**Keywords:** biological control of insect, decline, host plant effect, *Lolium multiflorum*, *Lolium perenne*, natural enemy, parasitism rate, pasture

## Abstract

Field parasitism rates of the Argentine stem weevil *Listronotus bonariensis* (Kuschel; Coleoptera: Curculionidae) by *Microctonus hyperodae* Loan (Hymenoptera: Braconidae) are known to vary according to different host *Lolium* species that also differ in ploidy. To further investigate this, a laboratory study was conducted to examine parasitism rates on tetraploid Italian *Lolium multiflorum*, diploid *Lolium perenne* and diploid hybrid *L. perenne* ×*L. multiflorum*; none of which were infected by *Epichloë* endophyte. At the same time, the opportunity was taken to compare the results of this study with observations made during extensive laboratory-based research and parasitoid-rearing in the 1990s using the same host plant species. This made it possible to determine whether there has been any change in weevil susceptibility to the parasitoid over a 20 year period when in the presence of the tetraploid Italian, diploid perennial and hybrid host grasses that were commonly in use in the 1990’s. The incidence of parasitism in cages, in the presence of these three grasses mirrored what has recently been observed in the field. When caged, weevil parasitism rates in the presence of a tetraploid Italian ryegrass host were significantly higher (75%) than rates that occurred in the presence of either the diploid perennial (46%) or the diploid hybrid (52%) grass, which were not significantly different from each other. This is very different to laboratory parasitism rates in the 1990s when in the presence of both of the latter grasses high rates of parasitism (*c*. 75%) were recorded. These high rates are typical of those still found in weevils in the presence of both field and caged tetraploid Italian grasses. In contrast, the abrupt decline in weevil parasitism rates points to the possibility of evolved resistance by the weevil to the parasitoid in the diploid and hybrid grasses, but not so in the tetraploid. The orientation of plants in the laboratory cages had no significant effect on parasitism rates under any treatment conditions suggesting that plant architecture may not be contributing to the underlying mechanism resulting in different rates of parasitism. The evolutionary implications of what appears to be plant-mediated resistance of *L. bonariensis* to parasitism by *M. hyperodae* are discussed.

## Introduction

Over the last 25 years there has been increasing confidence that the impact of the Argentine stem weevil (*Listronotus bonariensis*) on *Lolium*-based pasture grasses has declined (Goldson et al., 2014a,b, 2015). This has largely been based on the use of selected strains of *Epichloë* endophytes that confer pest resistance in ryegrass (Johnson et al., 2013) combined with the significant impact of the braconid parasitoid biological control agent, *Microctonus hyperodae* (e.g., Barker and Addison, 2006).

Recently, however, there has also been growing field evidence that *M. hyperodae* may be losing its efficacy as a biological control agent of *L. bonariensis*. This declining control has been based on reports of a notable reappearance of *L. bonariensis* damage to pasture (e.g., Popay et al., 2011). In response, and as part of an investigation into the loss of efficacy, research has been focused on comparing current weevil parasitism levels with those in the 1990s (e.g., Goldson et al., 2014a,b). Such data can be very variable due to fluctuations in weevil population dynamics and parasitoid oviposition activity. However, during *L. bonariensis* overwintering diapause and coinciding parasitoid diapause, parasitism rates remain constant due to the hiatus in the insects’ development (Goldson and Emberson, 1981; Goldson and McNeill, 1992). Such overwintering stability has therefore permitted meta-analyses of historical datasets, which have shown that parasitism rates have declined notably in *Lolium*-based pastures since the parasitoid’s initial establishment and equilibration in the first 6 years of its release (e.g., Goldson et al., 2014a,b).

It is tempting to attribute this downward trend in parasitism to weevil resistance arising from continuous and high parasitoid selection pressure over the last *c*. 20 years as has been discussed recently by Goldson et al. (2015). The prospect of resistance is supported by the fact that the parasitoid undergoes parthenogenic (thelytokous) reproduction while the weevil reproduces sexually. This situation is what is sometimes described as an ‘unequal evolutionary arms-race’. However, other mechanisms could also contribute to the decline, such as changed farming practice, climate change, and the use of novel endophytes. These possibilities have been variously investigated, thus far without identifying any clear causative reason for the parasitism decline (e.g., Goldson et al., 2015); thus the acquisition of rapidly evolved resistance remains a possibility.

Rapid evolution in insect biocontrol has been known to occur elsewhere. In a study of field crickets (*Teleogryllus oceanicus*) on the Hawaiian islands, Pascoal et al. (2014) showed that genetically based resistance in this species occurred twice and involved separate genetic changes on different islands within the archipelago. On both occasions the crickets stopped stridulating (after about 24 generations) because such activity attracted the parasitic fly (*Ormia ochracea*) and this species exerted negative selection pressure. *Listronotus bonariensis* may similarly have developed genetically based resistance as it has undergone *c.* 50 generations since the first releases of *M. hyperodae*.

It is possible that plant species used in pastures may play a part in the observed reduction in parasitism. Goldson et al. (2015) noted in the field that parasitism of *L. bonariensis* by *M. hyperodae* was significantly higher in tetraploid *L. multiflorum* (Italian) ryegrass paddocks than in diploid perennial (*L. perenne*) ryegrass paddocks. These perennial paddocks were exposed to the same *L. bonariensis* and *M. hyperodae* populations as the Italian paddocks. In order to see if such differences could also be detected in the laboratory, preliminary observations were made in 2014 and these suggested that weevils maintained on the Italian or on perennial plants had different parasitism rates (Goldson and Tomasetto, unpublished data).

Of the 23 different species of pasture plants now commercially available to farmers in New Zealand (Charlton and Belgrave, 1992; Charlton and Stewart, 1999), *Lolium perenne, L. multiflorum*, and the hybrid *L. perenne* × *L. multiflorum* (*L. boucheanum* syn. *Hybridum*) are the most common. A description of these three pasture plants can be found in Langer’s (1973) textbook.

This contribution describes a systematic laboratory study using grasses similar to those studied in the field by Goldson et al. (2015) to determine whether similar plant-associated differences in parasitism rates occurred in the controlled and very different environment of cages. At the same time, this also permitted direct comparison with those data obtained during similar and extensive laboratory-based parasitoid research and rearing throughout the 1990s (e.g., Goldson et al., 1993; McNeill et al., 1999, 2002). Through such comparison it was possible to determine whether, in the intervening years, there has been a reduction in laboratory weevil susceptibility to *M. hyperodae* similar to that which has been found in the recently collated extensive field parasitism data (e.g., Goldson et al., 2014a,b).

## Materials and Methods

### Grass Type and Parasitism Rate

The *Lolium* grasses used in this study were Italian tetraploid *L. multiflorum* (cv. Grasslands Tama), diploid *L. perenne* (cv. Grasslands Samson) and diploid hybrid *L. perenne* ×*L. multiflorum* (cv. Grasslands Manawa). For clarity and brevity these grasses are referred to as ‘Italian’ grass, ‘perennial’ grass and ‘hybrid’ grass, respectively; all were endophyte free. Endophytes were excluded because there is now a wide range of differently acting novel endophyte strains in use in New Zealand pasture grasses. Also endophytes may have subtle effects on parasitoid behavior although this has not shown up in a recent field study (e.g., Goldson et al., 2015). Finally endophytes often do not perform very well in the tetraploid *L. multiflorum* varieties, so in general it seemed prudent to exclude the endophyte variable from the experiment. The grass types that were chosen represent the typical pasture types used in New Zealand farming since the release of the parasitoid.

All experimental work was conducted at ambient laboratory temperatures (23 ± 2°C) and 16:8 L:D photoperiod. Weevil adults were collected from mid-Canterbury ryegrass pastures using a modified leaf blowing machine (Goldson et al., 2000) between January 11, 2016 and January 22, 2016. They were then purged of egg and larval parasitoids by storing them for a minimum of 40 days and a maximum of 55 days with the remaining unparasitised population used for the experiment. The *M. hyperodae* pupae that emerged from these weevils were reared to obtain adult parasitoids for this study. Overall as detailed below, the experiment comprised three main treatments (grass types) with two subtreatments (plant positioning). These were replicated four times. There we also four grass-free control cages making 28 cages in total.

The experiment was established on March 17, 2016 using 305 mm × 205 mm × 130 mm translucent plastic cages with gauze lids. The four grass main treatment replicates were the minimum required to deal with pseudoreplication (Johnson et al., 2016). All cages were stocked with 23 *L. bonariensis* and two *M. hyperodae.* Each cage contained one of the three grass species treatments in the form of two 150 mm long bouquets with their moistened roots and associated soil in tightly sealed small polythene bags at the base of the plants. This resulted in at least 40 tillers per box (Supplementary Figure S1). Each grass treatment comprised two subtreatments, in separate cages, whereby the bouquets were positioned either horizontally or vertically; thus the cages were, respectively, positioned either on one end, or lying flat (Supplementary Figure S1). This different positioning of the plants was specifically to gain an initial indication of whether departure from vertical plant architecture had an effect on weevil parasitism in any of the three grass types. Gerard (2000) noted that weevils tend to leave the upright foliage in the presence of the parasitoid and Phillips (2002) suggested that plant orientation in a cage may influence parasitoid efficacy. There was also a control treatment comprising four cages (two horizontal and two vertical) containing 23 *L*. *bonariensis*, two *M. hyperodae* and two water-soaked dental wicks to maintain humidity. All paired treatments were placed randomly in the laboratory (Supplementary Figure S1). Parasitoids were removed from the cages after 48 h. Thereafter the weevils were maintained in the same ambient conditions for another 3 days until March 22, 2016 when they were frozen at -20°C prior to being dissected to assess parasitism rates (i.e., number of parasitized weevils per total number of weevils dissected).

### Comparative Rates of Parasitism between the 1990s and 2016

In this study, the opportunity was taken to use the same *Lolium* grass types as were used throughout the 1990s during general research into *M. hyperodae* including the parasitoid’s mass-rearing for release (Goldson et al., 1993; McNeill et al., 1999, 2002). This allowed us to directly compare the results obtained from this experiment with both published and unpublished work conducted in the 1990s. Notably, while the exposure periods of the weevils in some of the comparator experiments were sometimes longer than 48 h this was of minor importance as Phillips et al. (1996) have shown that parasitoid ovipositional effort declines rapidly after the first 48 h.

### Statistical Analysis

To test for statistical significance between parasitism rates in the treatments and control, non-parametric complete random permutation tests (*n* cycles = 10000) were run for a one-way analysis of variance (ANOVA) via the package “*lmPem*” (Wheeler, 2010) and subsequently we tested the statistical significance via *post hoc* Tukey’s HSD pairwise permutation tests embedded in the package “*stats*” in R 3.2.1 (R Development Core Team, 2016). This approach implements the methods for permutation tests described by Kabacoff (2011).

## Results

### Grass Types and Parasitism Rates Within Foliage Positioning Subtreatments

**FIGURE 1:**
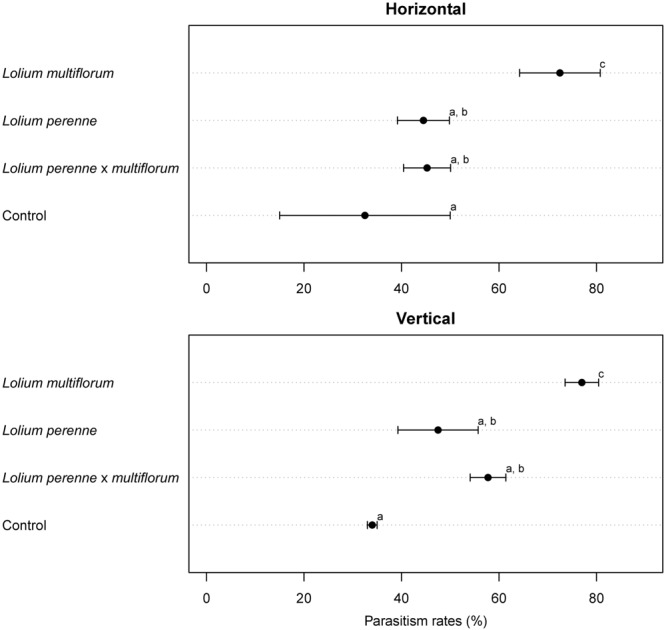
**Cleveland dotplot for *Microctonus hyperodae* mean parasitism rates (%) as measured in *Listronotus bonariensis* in cages containing Italian tetraploid *L. multiflorum* (cv. Grasslands Tama), diploid *L. perenne* (cv. Grasslands Samson) and diploid hybrid *L. perenne* × *L. multiflorum* (cv. Grasslands Manawa) and in cages containing no *Lolium* spp (Control).** The horizontal and vertical orientations (i.e., subtreatments) are shown here. Error bars represent SEM. Means with different letters were significantly different in pairwise comparisons.

The rate of parasitism of *L. bonariensis* by *M. hyperodae* in the presence of the experimental grass types and in the controls are presented in Supplementary Table S1 and the effects of grass type in **Figure 1**. In addition, **Tables 1–3** show these results in the context of other studies yielding parasitism rates both in the 1990s and recently, in the presence of the Italian, diploid, and hybrid grasses, respectively. For purposes of comparison, all data have been normalized to be expressed as the effect of one parasitoid per population of weevils.

**Table 1:** Summary table presenting the results of this study and other published and unpublished laboratory work on *Microctonus hyperodae* parasitism rates (% shown in bold) in caged *Listronotus bonariensis* populations in the presence of tetraploid *Lolium multiflorum* (cv. Grasslands Tama) plants.

Grass type and subtreatments	N° weevils	N° parasitoids	Duration (h)	Parasitism (%)	Reference	Date of experiment
***L. multiflorum***					
Horizontal treatment	30	1	216	**72**	McNeill et al., 1999	1999
Horizontal treatment	12	1	72	**67**	Goldson et al., 2004	2004
Horizontal treatment	10	1	48	**76**	Goldson and Tomasetto, unpublished data	2014
Horizontal treatment	12	1	48	**73**	This study	2016
Vertical treatment	10	1	48	**81**	Goldson and Tomasetto, unpublished data	2014
Vertical treatment	10	1	48	**77**	This study	2016

*Notably the 1990s data have been normalized as the number of weevils (N° weevils) per parasitoid (N° parasitoids). Also presented is the duration (hours) of the various laboratory experiments (Duration). The parasitism data used in this table comprised those from the both the horizontal and vertical grass subtreatments as the different orientations did not lead to significantly different results. Attack rates measured in the 1990s in cages with the tetraploid *L. multiflorum* plants did not differ significantly from more recent results.*

**Table 2:** Summary table presenting the results of this study and other published and unpublished laboratory work on *M. hyperodae* parasitism rates (% shown in bold) in caged *L. bonariensis* populations in the presence of diploid *Lolium perenne* (cv. Grasslands Samson) plants.

Grass type and subtreatments	N° weevils	N° parasitoids	Duration (h)	Parasitism (%)	Reference	Date of experiment
***L. perenne***					
Horizontal treatment	23	1	96	**68**	(Barker and Addison, 1996)	1992–3
Horizontal treatment	23	1	96	**80**	(Barker and Addison, 1996)	1992–3
Horizontal treatment	15	1	96	**73**	(Barker and Addison, 1997)	1994
Horizontal treatment	21	1	72	**94**	(Bultman et al., 2003)	2003
Horizontal treatment	10	1	48	**33**	Goldson and Tomasetto, unpublished data	2014
Horizontal treatment	12	1	48	**45**	This study	2016
Vertical treatment	10	1	48	**48**	Goldson and Tomasetto, unpublished data	2014
Vertical treatment	12	1	48	**45**	This study	2016

*Notably the 1990s data have been normalized as the number of weevils (No. weevils) per parasitoid. Also presented is the duration of the various laboratory experiments (Duration). The parasitism data used in this table comprised those from the both the horizontal and vertical grass subtreatments as the different orientations did not lead to significantly different results. Attack rates measured in the 1990s in cages with the diploid *L. perenne* plants were significantly higher than those from recent studies indicating the probability of acquired resistance by the weevils.*

**Table 3:** Summary table presenting the results of this study and other published and unpublished laboratory work on *M. hyperodae* parasitism rates (% shown in bold) in caged *L. bonariensis* populations in the presence of diploid hybrid *L. perenne* ×*L. multiflorum* (cv. Grasslands Manawa) plants.

Grass type and subtreatments	N° weevils	N° parasitoids	Duration (h)	Parasitism (%)	Reference	Date of experiment
***L. perenne* × *L. multiflorum***					
Horizontal treatment	23	1	72	**78**	(Barker and Addison, 1996)	1992–3
Horizontal treatment	7	1	48	**68**	(Barratt et al., 1996)	1994
Horizontal treatment	12	1	48	**45**	This study	2016
Vertical treatment	12	1	48	**58**	This study	2016

*Notably the 1990s data have been normalized as the number of weevils (No. weevils) per parasitoid. Also presented is the duration of the various laboratory experiments (Duration). The parasitism data used in this table comprised those from the both the horizontal and vertical grass subtreatments as the different orientations did not lead to significantly different results. Attack rates measured in the 1990s in cages with the diploid *L. perenne* plants were significantly higher than those from recent studies indicating the probability of acquired resistance by the weevils.*

#### Horizontal and Vertical Treatments Combined

The rate of parasitism in the presence of the Italian grass (75 ± 4%) was significantly higher than in either of the other grass treatments (*P* < 0.001). There was no significant difference in parasitism rates between cages containing perennial grass (46 ± 5%) and hybrid grass (52 ± 4%; *P* = 0.8). Parasitism rate in the control cages was 33 ± 7% and was significantly less than that found in cages containing grass (*P* < 0.01).

#### Horizontal Treatments

In the horizontal subtreatments, the rate of parasitism that occurred in the presence of Italian grass (73 ± 8%) was significantly higher (*P* < 0.001) than in either of the other horizontal subtreatments (**Figure 1**). There was no significant difference in parasitism rates between cages containing perennial grass (45 ± 5%) and hybrid grass (45 ± 5%, *P* = 0.08; **Figure 1**). Parasitism rate in the empty controls was 33 ± 18% which was not significantly different from the perennial and hybrid treatments but significantly less than in the Italian grass (*P* < 0.05; **Figure 1**).

#### Vertical Treatments

In the vertical subtreatments, the rate of parasitism that occurred in the presence of Italian grass (77 ± 3%) was significantly higher (*P* < 0.001) than in either of the other upright treatments (**Figure 1**). Again, there was no significant difference in parasitism rates in the cages containing the perennial grass (48 ± 8%) and the hybrid grass (58 ± 4%, *P* = 0.08; **Figure 1**). Parasitism in the empty control was 34 ± 1% which was not significantly different from the hybrid and perennial treatments (*P* = 0.5; **Figure 1**).

#### Horizontal versus Vertical Treatments

Horizontal versus vertical positioning of grass bouquets within the cages resulted in no significant differences in the rates of *L. bonariensis* parasitism by *M. hyperodae* across all of the grass types (Italian grass, *P* = 0.8; perennial grass *P* = 0.1, and hybrid grass = 0.8, respectively).

### Comparative Parasitism Rates between the 1990s and 2016

In the 1990s experiments, only horizontal treatments were used, therefore only the data from the horizontal subtreatments in this study were used for direct comparisons.

Descriptive analysis of the horizontal data in **Tables 1–3** show that in the 1990s, the mean parasitism rate in rearing cages containing perennial grass was 74 ± 4% as opposed to 39 ± 5% in the current study. Similarly the parasitism rate associated with the hybrid grass was 73 ± 6% in the 1990s compared with 45 ± 5% in this study. Conversely, the 73 ± 8% parasitism found in the presence of the Italian grass is very similar to that in the 1990s (70 ± 5%).

## Discussion

An emphasis of this study was to determine whether there have been significant changes in parasitism rates of Argentine stem weevil by *M. hyperodae* on typical pasture grasses since the 1990s rather than it being a definitive study of grass type effect on parasitism levels. Such direct comparison with historical data was possible because the same tetraploid Italian and hybrid cultivars were used in this study as throughout the 1990s. Thus any varietal genetic uncertainty is controlled for. The diploid *L. perenne* does not have complex genetic origins thus the direct comparison of cv. Samson in this study to cv. Nui in the 1990s is legitimate as both were derived from old New Zealand perennial pasture.

### Grass Types and Parasitism Rates

The observed 42% decline in *M. hyperodae* parasitism observed in this cage study in the diploid and hybrid grasses (**Tables 2** and **3**), compared to 1990s laboratory data, conforms to the findings of recent field-based studies that have indicated a similar *c.* 50% decline in parasitism rates in diploid grasses since the 1990s (Goldson et al., 2015). That such a reduction in parasitism did not occur in the presence of tetraploid Italian grass either in this study (**Table 1**) or in the field (Goldson et al., 2015) suggesting that whatever factor(s) reduced parasitism rates in the perennial and hybrid grasses (**Tables 2** and **3**) did not occur in the presence of Italian grass (**Table 1**). It is also significant that the laboratory parasitism rates in the Italian grasses in this study were typical of those previously occurring in both the perennial and hybrid grass types in the 1990s (**Tables 2** and **3**).

### Insignificant Plant Orientation Effects

Horizontal versus vertical positioning of grass bouquets within the cages resulted in no significant differences in the rates of parasitism of *L. bonariensis* across all of the grass treatments (**Table 1**; **Figure 1**). Such result indicates that, at least in the absence of soil or detritus, the orientation of plant material does not affect parasitism rates. This is contrary to Phillips (2002) suggestion that plant orientation in a cage may influence parasitoid efficacy. Further, the lower attack rates in the diploid and hybrid grasses were unlikely to have been based on the avoidance of parasitism by the weevils abandoning the foliage in the presence of the parasitoid as discussed by Gerard (2000). In the horizontal treatment, the grass leaves were broadly spread across the floor of the cage obviating the ability of *L. bonariensis* to drop off. The results of this study also point to the probable incorrectness of the contention of Goldson et al. (2015) who suggested that the higher levels of parasitism in the tetraploid *L. multiflorum* could have resulted from a difference in the architecture of the tetraploid versus diploid and hybrid perennial plants. This architecture hypothesis would seem to have been possible when considering vertical plants. However, the horizontal plant placement was a gross departure from the natural growth habit, yet there were no differences in the levels of parasitism between plant positioning subtreatments and the upright plants. This suggests that plant architecture was unlikely to be the underlying cause of the observed differences in parasitism between the grass types.

Finally, all treatments in the cages comprised grass bouquets that were bundled at their stem bases where the roots entered the polythene bags thereby providing limited scope for the weevils to ‘hide’ from the parasitoids. This is clearly different from the growth habit of the plants in the field.

### Ecological Implications

The lack of any notable difference in *L. bonariensis* parasitism rates in the cages containing diploid and endophyte-free hybrid grasses is significant ecologically. At the time of the first parasitoid releases, and in order to expedite its establishment by using areas with plentiful weevils, the work was conducted in either pure hybrid pastures or pastures comprising a mix of diploid and hybrid ryegrass (Goldson et al., 1998; Barker and Addison, 2006) as the hybrid is known to be preferred to the perennial as a host plant of the weevil (Goldson, 1982). As a consequence, some of the early parasitism field data were collected from these hybrid sites. This study has shown no differences in parasitism rates in the perennial and the hybrid grasses (Gaynor and Hunt, 1982; Goldson et al., 1998; Barker and Addison, 2006). This eliminates the prospect of any bias having occurred through possibly higher measured parasitism rates occurring in the limited and very early sampling in the hybrid grasses. This is consistent with the observation that high parasitism rates were typically found in the diploid pasture that surrounded the original release sites during investigation into the parasitoid’s lateral dispersal from the release sites (e.g., Barker and Addison, 1997; McNeill and Goldson, unpublished data).

### Mechanisms for the Measured Differences in Attack Rates in the Laboratory and the Field

The attack rates measured in this caging study were very similar to those currently observed in the field (Goldson et al., 2015). This is surprising given the obvious environmental differences between the field and laboratory cages (e.g., no soil or detritus).

It can be hypothesized that the underlying mechanism for the observed general decline in parasitism rates since the 1990s (e.g., Goldson et al., 2014a,b) could have been based on the adoption of novel endophytes. However, none of the grasses in this laboratory study were infected with endophytes. Additionally, Goldson et al. (2015) in a 5-month summer field study, showed no significant field effects of endophyte on *L. bonariensis* parasitism rates in the mix of *Lolium* varieties and endophytes.

Contrary to the findings here, the data collected in the 1990s indicated no differences in parasitism rates, irrespective of grass type. At that time weevil parasitism rates in the hybrid and perennial grasses were comparable to those now only found in the tetraploid Italian plants (**Tables 1–3**).

Barker (1989) observed much higher rates of *L. bonariensis* feeding and oviposition in the leaves of tetraploid Italian grasses than in the perennial grasses. Related to this Phillips (2002) showed that weevil feeding, walking, grooming defecating, or mating predisposes it to higher levels of parasitoid attack and this therefore could be the reason for higher parasitism rates on the Italian grass. Conversely, Barker (1989) also showed that hybrid ryegrass (cv. Grasslands Manawa) is equally favored as a host by *L. bonariensis* as the Italian grass. In spite of this, the results here showed significantly less parasitism in the hybrid grasses than in the Italian grasses. This observation suggests that the intensity of weevil feeding and oviposition *per se* may not entirely be the reason for varied parasitism rates. Significantly, the growth habit of the hybrid grass is much closer to that of the diploids and neither of these ryegrass types support the same levels of leaf-feeding and oviposition as found in the Italian plants (Barker, 1989). It is also of interest that parasitism rates in grass-free control cages, while usually lower than in the cages with the grasses present, still showed substantial parasitism indicating that *L. bonariensis* remains susceptible to parasitism when not feeding or ovipositing.

The decline in parasitism in the hybrid and diploid grasses since that 1990s has not coincided with any sign of physiological resistance in the weevils. In spite of 1000s of weevils having been dissected by numerous workers since the introduction of the parasitoid, there has never been any observation of *M. hyperodae* early stages being encapsulated in *L. bonariensis* (e.g., Goldson et al., 2015).

### Adaptive Implications

In general, the results in this study support the contention of Goldson et al. (2015) that if selection pressure has led to an enhancement of some kind of parasitoid-avoiding behaviors amongst *L. bonariensis*, then such evolution would most likely to have occurred in the country’s extensive diploid pastures rather than in the rare tetraploid Italian *L. multiflorum* pastures (B.R. Belgrave, Grasslanz Technology Ltd., pers. comm.).

## Conclusion

It has been confirmed that different patterns of parasitism associated with different *Lolium* species and ploidy observed in the field also occurred in the laboratory experiments. At the same time, it has been demonstrated in the laboratory that diploid *L. perenne* and the diploid hybrid *L. perenne* ×*L. multiflorum* no longer support the levels of attack that were found in the 1990s. This is consistent with the contention that the weevil has evolved resistance to the parasitoid. The cause and mechanisms of this have yet to be determined; for example it is not known if there is a species or a ploidy effect, although field work has shown that parasitism levels in tetraploid *L. perenne* are no different from those in diploid *L. perenne* (Goldson et al., 2015). The possibility that resistance to a biological agent is dependent on plant type would seem to be unique in the literature.

There is now the prospect genetic and genomic analysis of both the weevils and parasitoid to explore further the underpinning of the observations in this contribution. By combining various approaches, the understanding of the reasons for success and failure in biological control must continue to develop (Mills and Kean, 2010).

## Author Contributions

SG and FT conceived and designed the experiment. FT performed the analysis. SG and FT wrote the article with significant intellectual input from both authors. FT and SG conducted the experimental work described in the article with SG overseeing collection of further data used in **Tables 1–3**. Both authors contributed to the discussion and approved the final manuscript.

## Conflict of Interest Statement

The authors declare that the research was conducted in the absence of any commercial or financial relationships that could be construed as a potential conflict of interest.
