# Supplementary material for: Apparent Acquired Resistance by a Weevil to Its Parasitoid Is Influenced by Host Plant
Source: Front Plant Sci. 2016 Aug 23;7:1259. doi: 10.3389/fpls.2016.01259 (PMC4994474; doi:10.3389/fpls.2016.01259)
Supplement: Supplementary file 1 [file Table_1.DOCX]

**SUPPLEMENTARY MATERIALS**

**Table S1** Summary table of *Microctonus hyperodae* mean parasitism rates (%) and standard errors (SEs) as measured in *Listronotus bonariensis* in cages containing Italian tetraploid *L. multiflorum* (cv. Grasslands Tama), diploid *L. perenne* (cv. Grasslands Samson) and diploid hybrid *L. perenne* × *L. multiflorum* (cv. Grasslands Manawa) and in cages containing no *Lolium* spp. bouquets (Control) on 22 March 2016. The subtreatment effects (i.e horizontal or vertical bouquets) are also shown.

| **Grass type and subtreatments** | **Parasitism (%)** | **SE (%)** |
| --- | --- | --- |
| **Tetraploid *Lolium multiflorum*** | | |
| Horizontal | **73** | **8** |
| Vertical | **77** | **3** |
| **Diploid *Lolium perenne*** | | |
| Horizontal | **45** | **5** |
| Vertical | **48** | **8** |
| **Hybrid *L. perenne* × *L. multiflorum*** | | |
| Horizontal | **45** | **5** |
| Vertical | **58** | **4** |
| **Control** | | |
| Horizontal | **33** | **18** |
| Vertical | **34** | **1** |

**Figure S1** Example of one of the randomly placed replicates (n_total_ = 28) in the laboratory experiment established on 17 March 2016 using translucent plastic cages with gauze lids. All cages were stocked with 23 *Listronotus bonariensis* and two *Microctonus hyperodae.* In each cage was also placed one of the three nil endophyte grass treatments [Italian tetraploid *L. multiflorum* (cv. Grasslands Tama), diploid *L. perenne* (cv. Grasslands Samson) and diploid hybrid *L. perenne* × *L. multiflorum* (cv. Grasslands Manawa)] in the form of two 150 mm bouquets that were positioned either horizontally or vertically. There were also control cages (n_total_ = 4; two of which were horizontal and two vertical) where instead of the grass two water-soaked dental wicks were placed to maintain humidity.
