# Supplementary figures and images for: Apparent Acquired Resistance by a Weevil to Its Parasitoid Is Influenced by Host Plant
Source: Front Plant Sci. 2016 Aug 23;7:1259. doi: 10.3389/fpls.2016.01259 (PMC4994474; doi:10.3389/fpls.2016.01259)

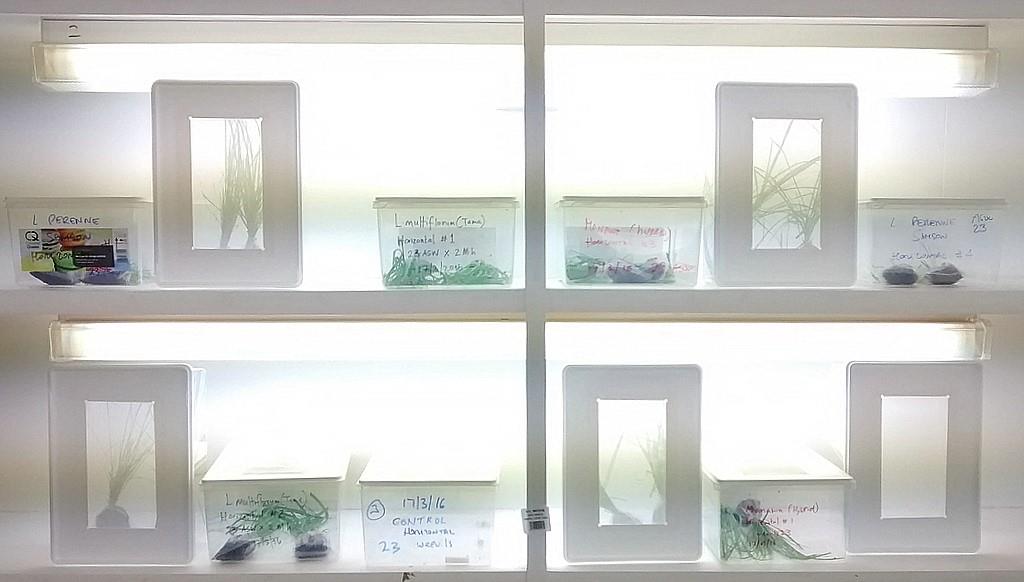

Supplement: Supplementary file 2 [file Image_1.JPEG]
